# Empty Beds in London Hospitals?

**Published:** 1908-12-19

**Authors:** 


### HOSPITAL ADMINISTRATION.

### CONSTRUCTION AND ECONOMICS.

### EMPTY BEDS IN LONDON HOSPITALS?

A gentleman whose identity it is not difficult to place has written three letters to the *Times* signing himself "Economy." The first letter appeared on October 29 and attracted little attention. A second letter, therefore, appeared from him in the *Times* on November 14, which was replied to by Mr. Danvers Power. On November 28 "Economy" reiterated his statements, and it is well that the Distribution Committee of the King's Fund have examined and disposed of them in their report which was presented to the Council at its meeting at Marlborough House on the 14th instant. "Economy" is not fair, having apparently all the facts readily available, in not producing, as he was challenged to produce, a list of the hospitals where he claims there are empty beds. "Economy" maintains that according to the hospitals' own returns there are a number of beds vacant for want of funds, and that proposals are being made to provide additional beds. As to the first point, the vacant beds, the Distribution Committee are careful to point out that although the capacity of some hospital buildings may represent a total accommodation much in excess of the actual average number of beds occupied, it does not follow that such hospitals in reality contain many or any vacant beds in the sense that those beds could be made available for patients if funds were forthcoming to support them.

The Committee have, however, carefully considered every case in which the existence of vacant beds occurs in any institution applying to the Fund for a grant. The Committee as in duty bound excludes from this category beds which are temporarily unoccupied merely as the result of the periodic changes of occupants, the provision of a surplus for accidental emergencies, and the occasional closing of wards for necessary cleaning. All these governing factors "Economy" ignores. As the result of their careful investigation the Distribution Committee find that most of the beds reported as vacant consist of four classes, either of: (*a*) beds which are at present used for the accommodation of nurses or for other necessary hospital purposes which could not be provided for elsewhere without expenditure on building; (*b*) beds the accommodation for which was erected without due consideration of the local demand or the prospect of maintenance, and which have never been opened for use; (*c*) beds which have recently become available as the result of extensions, and which there is at present no reason to suppose the hospitals will not be able to open as public support (including the normal grants from the King's Fund) increases in response to the increased demand; and (*d*) beds in hospitals which have for one reason or another failed to secure or to retain public confidence. When these four classes of beds are excluded, as they ought to be excluded in considering on its merits the contention of "Economy," the Distribution Committee report that the only vacant beds left which may properly be described as unoccupied for want of funds amount to less than 25, and even in regard to these 25 or some of them, there are special circumstances which render their availability doubtful. The Distribution Committee thus effectually dispose of the statements made by "Economy" and are in our judgment wise in the decision they arrive at. This is that, in view of the existing distribution of the demand for hospital accommodation, the resources of the King's Fund can at present be better employed in assisting the extension of the outlying hospitals situated in or near to the poorer districts remote from the larger general hospitals, than in opening the beds reported as vacant. It is much to be regretted that "Economy" should have persisted in urging his individual view, which is thus shown to rest on no solid foundation, against the considered judgment of the men who from fulness of knowledge at present direct so successfully the formation of public opinion in regard to the requirements of the voluntary hospitals.

Having regard to the population and the actual number of hospital beds available, within certain districts of the metropolitan area, there can be no question that every effort should be made by the great central funds, and by those who are willing and able to give liberal financial support to the hospitals, to place the authorities of the Miller Hospital, Greenwich, the Bolingbroke Hospital, Wandsworth, the Prince of Wales's General Hospital, Tottenham, and the Great Northern Central Hospital, Holloway, in a position of sufficient financial strength, to extend and complete the bed accommodation required by these districts, to the extent they have formulated in the appeals and papers which they have recently issued. It is a great hardship and an unnecessary expense not to provide the poor of the outlying and poorer districts of the Metropolis with adequate hospital accommodation of the best kind in immediate juxtaposition to their own homes. The sooner this fact is recognised and acted upon the better. We cordially commend the appeals of the four institutions we have mentioned to the earnest and liberal support of large donors, especially, at the present time.

